# Web-Based Asynchronous Teleconsulting for Consumers in Colombia: A Case Study

**DOI:** 10.2196/jmir.9.4.e33

**Published:** 2007-10-22

**Authors:** José Ignacio Valenzuela, Arturo Arguello, Juan Gabriel Cendales, Carlos A Rizo

**Affiliations:** ^2^Centre for Global eHealth InnovationUniversity of TorontoTorontoCanada; ^1^Centro de Educación Virtual y Simulación e-HealthDivisión de EducaciónFundación Santa Fe de BogotáBogotáColombia

**Keywords:** Case study, Colombia, consultation, remote, eHealth, teleconsultation, telemedicine

## Abstract

**Background:**

Fourteen years after the reform to Colombia’s health system, the promises of universality, improved equity, efficiency, and better quality of care have not materialized. Remote areas remain underserved and access to care very limited. Recognizing teleconsultation as an effective way to improve access to health care and health information, a noncommercial open-access Web-based application for teleconsultation called Doctor Chat was developed.

**Objective:**

The objective was to report the experience of the Center for Virtual Education and Simulation eHealth (Centro de Educación Virtual y Simulación e-Salud) with open-access Web-based asynchronous teleconsultation for consumers in Colombia.

**Methods:**

A teleconsultation service in Spanish was developed and implemented in 2006. Teleconsultation requests were classified on three axes: (1) the purpose of the query, (2) the specialty, and (3) the geographic area of the query. Content analysis was performed on the free-text queries submitted to Doctor Chat, and descriptive statistics were gathered for each of the data categories (name, email, city, country, age, and gender).

**Results:**

From September 2006 to March 2007, there were 270 asynchronous teleconsultations documented from 102 (37.8%) men and 168 (62.2%) women. On average, 1.4 requests were received per day. By age group, the largest number of requests (n = 80; 30%) were from users 24-29 years, followed by users (n = 66; 24%) 18-23 years. Requests were mainly from Colombia (n = 204; 75.6%) but also from Spain (n = 17; 6.3%), Mexico (n = 11; 4.1%), and other countries. In Colombia, 137 requests (67.2%) originated in Bogotá, the nation’s capital, 25 (12.4%) from other main cities of the country, 40 (19.7%) from intermediate cities, and 2 (0.7%) from remote areas. The purpose of the majority of requests was for information about symptoms, health-related problems, or diseases (n = 149; 55.2%) and medications/treatments (n = 70; 25.9%). By specialty, information was most requested for gynecology and obstetrics (n = 71; 26%), dermatology (n = 28; 10%), urology (n = 22; 8%), and gastroenterology (n = 18; 7%), with anesthesiology, critical care, physical medicine and rehabilitation, and pathology being the least requested (n = 0; 0%). Overall, sexual and reproductive health (n = 93; 34%) issues constituted the main query subject. The average time to deliver a response was 120 hours in 2006 and 59 hours in 2007. Only 19 out of 270 users (7%) completed a survey with comments and perceptions about the system, of which 18 out of 19 (95%) corresponded to positive perceptions and 1 out of 19 (5%) expressed dissatisfaction with the service.

**Conclusion:**

The implementation of a Web-based teleconsulting service in Colombia appeared to be an innovative way to improve access to health care and information in the community and encouraged open and explicit discussion. Extending the service to underserved areas could improve access to health services and health information and could potentially improve economic indicators such as waiting times for consultations and the rate of pregnancy among teenagers; however, cultural, infrastructural, and Internet connectivity barriers are to be solved before successful implementation can derive population-wide positive impacts.

## Introduction

Colombia’s health system, called General Social Security System for Health (Sistema General de Seguridad Social en Salud, SGSSS) is a mixed system (partially publicly funded and partially privately subsidized). Although major improvements have been achieved since the reform in 1993, the promises of universality, improved equity, efficiency, and better quality of care have not materialized [1].

In spite of the ascending trend over the past years in SGSSS’s overall population coverage (from 36% in 2000 to 74.1% in 2005) [2] and the doubling in the number of medical specialists in the past decade [3], many agree that the reform has increased the inequity in the allocation of resources, the access to health services, and the distribution of spending on health [4]. This is further exacerbated by the inequitable distribution of specialists throughout the country, as most concentrate in the main cities (61.5% of the total number of specialists are located in the four main cities) [3], thus contributing to the focalized excess of supply in places where the latest technology and the highest quality-of-care standards are available and leaving the remote and rural areas of Colombia “unprotected.” While SGSSS’s inequities are not to be fully solved in the short term and a national redistribution of specialists is unlikely, solutions need to be provided quickly.

Almost a decade after the beginning of the new millennium, great attention has been drawn to the application of the emerging information and communication technologies in the health care setting, and health informatics has received recognition as a fundamental strategic component for achieving the greatly desired Global Health Development as stipulated by the “Health for All in the 21st Century” strategy of the World Health Organization (WHO) [5]. Furthermore, making “available the benefits of new technologies, especially information and communications” [6] is embedded as one of the targets to achieving the Health and the Millennium Development Goals established by the WHO. Among the potential applications of these new technologies, teleconsultation has been reported as an effective way to improve access to health care and health information [7], and its use has been encouraged: “Access to care should be provided over the Internet.... Instead of a $65 office visit and half-day off work, a 2-minute email communication could meet many patients’ needs more responsively and at lower cost” [8].

**Figure 1:**
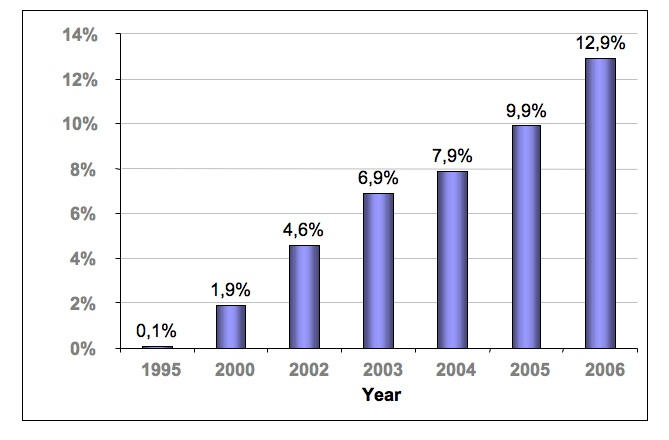
Internet penetration in Colombia [9,10]

In Colombia, the Internet promises to play a crucial role in health care delivery as usage continues to grow. Penetration has steeply risen from 4.6% in 2002 [9] to 12.9% in 2006 [10] (Figure 1), and access has become a national policy [11]. Additionally, broadband prices have continuously dropped over the past years and infrastructure (installed capacity) has pervasively increased [12]; in fact, in 2005, the country occupied fourth place in terms of broadband growth worldwide and first place among Latin America, with a 151% increase in the number of users with respect to the previous year [12]. 

In this context, and with the aim of providing a tool that could serve as a basis for improving access to health care services in the Colombian community by exploring the potential that new technologies can offer to populations in-need, such as those of developing countries, a noncommercial Web-based application for teleconsultation called Doctor Chat [13] was developed at the Center for Virtual Education and Simulation (Centro de Educación Virtual y Simulación, División de Educación, Fundación Santa Fe de Bogotá [14]) and was implemented in September 2006. Six months after going live, we hereby present our experience with asynchronous teleconsultation in Colombia.

## Methods

### Nature, Design, and Development of Doctor Chat

Doctor Chat was designed and developed as an open-access free teleconsultation service in Spanish, using a user-centered approach by which needs assessment, interface configuration, and prototyping were conducted by the teleconsultation service’s multidisciplinary team of two physicians, one graphic designer, and one programmer (who also acts as the Web administrator), taking as the baseline referent applications developed elsewhere [15,16]. Target users were established as “the general community of Colombia, especially those located in remote and underserved areas.”

Doctor Chat is composed of a Web-based application structured as a series of HTML pages created by a Web server (Red Hat Enterprise Linux 4) to store and retrieve data in a relational database (MySQL version 5.0.24-standard). The application can be accessed by the general public over the Internet using any Web browser, and it incorporates a synchronous and an asynchronous teleconsultation tool.

### Asynchronous Teleconsultations With Doctor Chat

**Figure 2:**
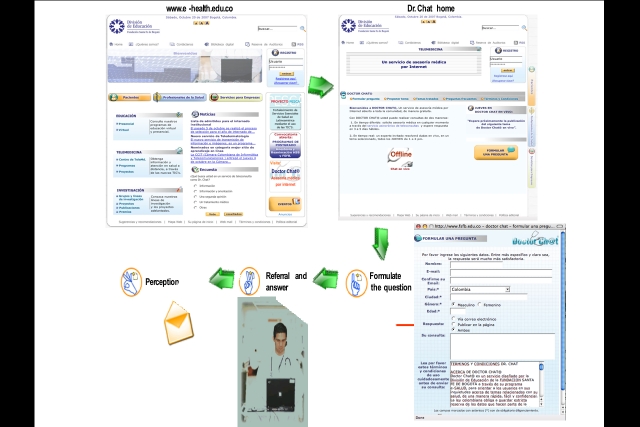
Doctor Chat: how does it work?

Users of Doctor Chat enter the Center’s Web page [14] and click on the link “Doctor Chat” to be directed to the teleconsultation service’s home page, which contains general information, indications and mode of use, the specific thematic areas covered in the past (with specialists’ answers to common concerns), the date and time of the next synchronous session, the guest specialist, and other relevant information. By clicking on the “Formulate a question” (“Formular una pregunta”) icon on the right side of the screen (or the homologous link on the left side of the screen), users are presented the format for asynchronous teleconsultation. The format includes several data fields: (1) basic demographic information, (2) mode of response (whether users prefer to have the answer sent to their personal email, published on the asynchronous forum, or both), and (3) “Make your consultation" (“Su consulta:”), where users formulate their question in a blank limitless cell. In order to protect user confidentiality, only the “Make your consultation,” “I accept and understand the Terms and Conditions,” and “Select your preferred mode of response” fields are required. Other information is not mandatory, and the email field is only required when the user selects to have the response sent to his/her email account (Figure 2).

Regardless of the preferred mode of response selected by the user, and in addition to the automatic publication of the question in the asynchronous forum (when requested by the user), each question is automatically directed to a centralized Doctor Chat email account (to which the two physicians and the Web administrator have access) and to the institutional personal email accounts of the two physicians. Only Doctor Chat’s medical team, composed of a senior doctor (a specialist in internal medicine) and a junior doctor (general practitioner), has access to the questions posed. Additionally, a relational database is automatically fed after submission of each question. Along with the response, a single-question informal survey of user’s satisfaction is sent (“Are you satisfied with Doctor Chat’s service? Please send us your comments to improve the service”).

### Data Analysis

Content analysis was performed on the free-text queries submitted to Doctor Chat. Requests were classified according to three schemes: (1) the purpose of the query, (2) the specialty, and (3) the geographic area of the query. A taxonomy of patient requests proposed by Kravitz et al [17] was used to categorize the purpose of the query, and each request was classified according to the topic into one of the 29 specialties available at our institution.

Descriptive statistics were gathered for each of the data categories (name, email, city, country, age, and gender) and the location from which each query was submitted was determined by the response to the “country” cell. 

## Results

Doctor Chat went live on September 15, 2006. From that date to March 22, 2007, 270 teleconsultations from 102 (37.8%) men and 168 (62.2%) women of all age ranges were received (Table 1). There were 130 (48.1%) users between September 15 and December 31, 2006, and 140 (51.9%) users between January 1 and March 22, 2007, for an average of 1.2 teleconsultations per day during 2006, 1.7 during 2007, and a consolidated average of 1.4.

On average, each of the responses sent by Doctor Chat’s medical team contained 215.4 words (range: 39-832 words). The average time for each response was 120 hours (5 days) during 2006 and 59.04 hours (2.46 days) in 2007.

As selected by all the users, all responses were sent to the email addresses provided in the request.

Among the 270 users of Doctor Chat, 19 (7%) voluntarily replied with their comments and perceptions; 18 of these (95%) were positive perceptions, whereas 1 (5%) expressed dissatisfaction with the service.

**Table 1:** Gender and ages of Doctor Chat users

	Number	Percentage
**Gender**		
Female	168	62.2
Male	102	37.8
**Age** (years)		
< 18	13	4.8
18-23	66	24.4
24-29	80	29.6
30-35	34	12.6
36-40	24	8.9
41-45	14	5.2
46-50	15	5.6
> 50	15	5.6
N/A^*^	9	3.3
**Total**	**270**	**100**

^*^N/A, no answer.

### Purpose of the Requests

The average number of words per consultation was 48.81 (range: 4-306). On average, each of these consultations contained only one request. By taxonomical category (as described by Kravitz et al [17]), the majority were requests for information about symptoms, problems, or diseases (n = 149; 55.2%) (Table 2); among these, 19 (12.8%) users particularly requested a second opinion while the other 130 (87.2%) searched for general information.

Among requests for information, 70 (25.9%) consultations belonged to the medications/treatments subcategory; 58 (82.9%) inquired for information about any type of treatment; 7 (2.6%) requested information for prevention of disease, mainly acute myocardial infarction and cancer; 5 (7.1%) asked for information regarding a surgical procedure that the user was to undergo. Another 5 (7.1%) requested information on nonconventional treatments (alternative medicine), and 2 (2.9%) asked for information regarding postsurgical recommendations.

Among the 12 (4.4%) requests that were classified within “other request for information,” 8 (66.7%) searched for data to complete homework or academic assignments and the remaining 4 (33.3%) inquired on how to become an organ donor.

Among requests for action (see Table 2), specifically in the medications/treatments subcategory, most patients asked about contraceptive methods, diets, and treatment for a variety of infections.

**Table 2:** Requests by taxonomical category

		Number (%)
**Request for information**		**251 (93)**
	Symptoms, problems, or diseases	149 (55.2)
	Psychosocial problems	0 (0)
	The physical examination	0 (0)
	Test or diagnostic investigations	7 (2.6)
	Medications/treatments	70 (25.9)
	Prevention	7 (2.6)
	Index physician-patient relationship	0 (0)
	Other physicians	1 (0.4)
	3rd party payer or managed care issues	0 (0)
	Other administrative issues	5 (1.9)
	Other request for information	12 (4.4)
		
**Request for action**		**19 (7)**
	Physical examination	0
	Laboratory test, x-rays, or other study	0
	Referral to other physician	2 (0.7)
	Referral to nonphysician	0
	Medication/treatments	17 (6.3)
	Administrative action: 3rd party payer	0
	Administrative action: other	0
	Other request for action	0

### Requests by Specialty

By specialty, requests fell mainly into three areas, in descending order of frequency (Table 3):

1. Sexual and reproductive health (91 requests, 34%)

- All 71 questions (78% of the sexual and reproductive health questions; 26% of total requests) that related to gynecology and obstetrics concerned sexual and reproductive health. Among these, 26 (36%) corresponded to contraception methods, 9 (13%) concerned fetal abnormalities during pregnancy, and 36 (51%) made reference to sexually transmitted infections (STIs).
- Among the 22 questions (8% of total requests) related to urology, only 20 (91%) concerned sexual and reproductive health; specifically, these 20 requests inquired about STIs, whereas the other 2 (9%) inquired about prostate cancer and unstable bladder.
- Among the 56 (62%) requests related specifically to STIs, 8 (14%; 3% of total requests) asked about HIV/AIDS.
- Of the overall 91 (34%) requests regarding reproductive health, 32 (35%) were formulated by users in the 18-23 year age group, 30 (33%) in the 24-29 age group, 15 (16%) in the 30-40 group, 8 (9%) in the over 40 group, and 6 (7%) by users younger than 18 years.

2. Dermatology

- There were a total of 28 requests (10%) of which 20 (71%) asked for information regarding removal of striae, moles, scars, or tattoos; 3 (11%) inquired about treatment of acne; 5 (18%) asked about suspected malignant lesions and skin cancer.
- Of the 28 dermatology requests, 17 (61%) came from females, and 11 (39%) came from males.

3. Gastroenterology

- The 18 (7%) requests for information on gastroenterology specifically concerned irritable bowel syndrome, gastroesophagic reflux, gastritis, acute gastroenteritis, and peptic ulcer.
- Users who formulated gastroenterology requests were from both genders and were dispersed among all age groups.

**Table 3:** Requests by specialty

Specialty	Number	Percentage
General medicine	14	5.2
Surgery	4	1.5
Transplants and organ donation	8	3.0
Orthopedics	8	3.0
Otorhinolaryngology	2	0.7
Plastic Surgery	2	0.7
Urology	22	8.1
Gynecology and obstetrics	71	26.3
Ophthalmology	3	1.1
Anesthesiology	0	0.0
Internal medicine (general)	7	2.6
Cardiology	11	4.1
Endocrinology	6	2.2
Gastroenterology	18	6.7
Nephrology	1	0.4
Neumology	1	0.4
Dermatology	28	10.4
Hematology	1	0.4
Neurology	7	2.6
Rheumatology	1	0.4
Non-traditional medicine	2	0.7
Oncology	3	1.1
Toxicology and psychoactive substances	2	0.7
Critical care	0	0.0
Pediatrics	13	4.8
Physical medicine and rehabilitation	0	0.0
Psychiatry	7	2.6
Diagnostic imaging	1	0.4
Nutrition	6	2.2
Oral health	5	1.9
Pathology	0	0.0
Others	16	5.9
**Total**	**270**	**100.0**

Among the overall 270 consultations, 7 (2.6%) described emergency-related symptoms: 5 (1.9%) concerned chest pain and 2 (0.7%) denoted pediatric emergencies. Response to these requests was prioritized, and patients were advised to attend an emergency department immediately upon reading the response. These types of requests were given a response in less than 24 hours.

None of the users chose to have their requests published in the asynchronous discussion forum.

### Origin of the Requests

Three quarters of consultations were initiated in Colombia, but Doctor Chat received inquiries from several other countries, including Aruba, Spain, and the United States (Table 4).

**Table 4:** Requests by country

Country	Number	Percentage
Colombia	204	75.6
Spain	17	6.3
México	11	4.1
N/A	7	2.6
Argentina	6	2.2
Perú	6	2.2
United States	4	1.5
Venezuela	4	1.5
Chile	3	1.1
Bolivia	2	0.7
Ecuador	2	0.7
Aruba	1	0.4
Panamá	1	0.4
Paraguay	1	0.4
Uruguay	1	0.4
**Total**	**270**	**100**

In Colombia, most consultations originated in Bogotá (n = 137; 67.2%) and the other four main cities of the country (n = 25; 12.4%); only 2 (0.7%) requests came from remote areas, and the remaining were from intermediate cities (n = 40; 19.7%).

## Discussion

### Implementation of Doctor Chat

As in many other developing countries, access to adequate health services in Colombia is suboptimal, especially in rural and remote areas. While the main cities have ample infrastructure, the latest technology, and high quality-of-care standards, distant areas of the country are notably underserved.

A number of strategies have been proposed to improve access to health care in order to [18]

- enlarge the capacity overall (eg, increasing entry to medical schools and providing financial or other incentives to physicians to become general practitioners)
- maximize the output of existing resources by promoting the formation of multidisciplinary teams to increase access
- distribute resources to underserved areas to address inequalities in access
- improve specific aspects of access such as waiting times and continuity of care

In Colombia, however, as entry to and graduation from medical school is unregulated, there currently exists an excess of supply of physicians. Nevertheless, the national distribution of the medical workforce is unequal. Moreover, incentives to promote redistribution (general practitioners versus specialists, and location of qualified doctors) have been insufficient to motivate a significant number of professionals to migrate to remote areas.

In this context, the implementation of a teleconsultation service appeared to be an innovative way of delivering health care and advice in Colombia. However, we were aware that important barriers such as limitations in connectivity (79.8 Internet users per 1000 inhabitants) [19], “informatic illiteracy,” and lack of an “IT culture”, mainly in distant areas of the country, would make the implementation highly cumbersome in terms of usage. Nonetheless, Doctor Chat was timidly established as an “experimental” application to evaluate the impact and potential of a new tool to improve access to health information in the local community. As far as we know, Doctor Chat is the first free open-access Web-based teleconsultation service in Colombia.

Even though no publicity was put in place to encourage its use, in only 6 months a high number of requests have been received, relative to the time elapsed, compared to reports from countries with higher levels of connectivity [20-24]. Yet, of the 204 (75.6%) consultations from Colombia, about 187 (92%) came from cities with third/fourth-level health institutions, and 157 (77%) of the requests were received from the four main cities of the country. Surprisingly, among the seven geographic departments that are considered a national priority for implementation of telemedicine services in Colombia [25], Doctor Chat received two requests from one of these regions. Nevertheless, our results do not allow us to draw conclusions about the impact of the teleconsultation application in remote and underserved areas.

**Figure 3:**
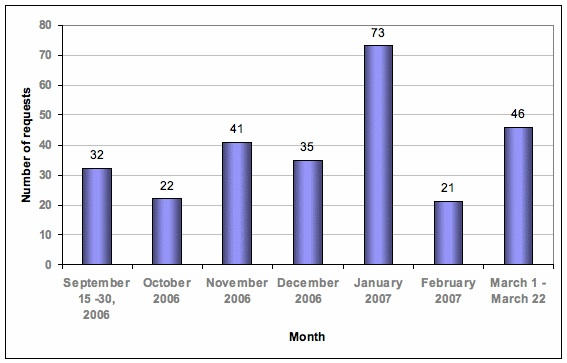
Use of Doctor Chat: number of requests by month

Eysenbach has proposed the law of attrition, which states that in eHealth trials which start with a fixed number of users, usage will decrease because “a substantial proportion of users drop out before completion or stop using the application” [26]. As our user base is variable with new users entering and old users dropping out, our utilization rates vary greatly from month to month (Figure 3). Nonetheless, no final conclusions can be derived from these observations, which may change after publicity and expansion of the teleconsultation service are put in place and a more representative time interval has passed.

### General Results

It has been suggested that possible reasons for patients using the Web in search of health advice include (1) frustration from insufficient or low-quality information previously received (from treating physicians or other sources), (2) failure of previous therapies, (3) preference to remain anonymous, and (4) lack of availability of health services  [27]. Similar to the results reported by Kravitz et al [17] and Sittig [28], and possibly as a consequence of the reasons listed above, most (93%) of the consultations were requests for information, compared to requests for action (7%). However, and in contrast to the Kravitz et al [17] and Sittig [28] studies, the most common information requests involved queries regarding symptoms, problems, or diseases (55.2%), followed by information about medications or treatments (25.9%). Because access to health care services is available in the main cities of Colombia, from where most requests were received, this observation cannot be explained on the basis of user remoteness. Because waiting times for a face-to-face consultation can be extensive and the administrative process, cumbersome, the explanation could lie in users’ desire to assess the severity of their symptoms before attempting to arrange a face-to-face consultation. In this manner, and on a wider scale, the teleconsultation service could play a role in adequately filtering and triaging patients, hence reducing waiting times and clearing congestion of the health system.

By medical specialty, the majority of requests concerned sexual and reproductive health issues, and many contained direct and explicit content; additionally, most users decided to use nicknames to remain anonymous. In this context, and taking into account the general characteristics of the local culture in which sexual-related subjects are still considered taboo, we believe the teleconsultation service encouraged open questioning and facilitated discussion, as the nature of many of the questions posed could have been perceived as socially inappropriate. Furthermore, as most (35%) requests regarding sexual and reproductive health were formulated by users between 18 and 23 years of age, observing the trend over time could be valuable in establishing education campaigns and supporting an exclusive Web-based discussion forum on this theme, targeted at high schools and colleges. This is important in the context of Latin American countries like Colombia and Brazil, where national-level sexual education failures have been reported and pregnancy rates among teenagers continue to rise, thereby increasing poverty among the regions. “Each year of [sexual] education reduces the probability of pregnancy before 20 years of age by 2%” (cited in Spanish in [29]).

Interestingly, we did not encounter any user whose reason for consulting was remoteness or lack of access to health services. As far as we can tell, users came principally from the main cities of Colombia, which have excellent health facilities. This may, to a certain extent, be a reflection of the limited access to the Web in distant areas of the country. Regarding how patients found us and why they would be interested in using our service, we suppose this could have been the result of our high institutional ranking in the country (the second health institution among the top 300 of the country) [30] and our privileged position in search engines such as Google [31].

In summary, our experience with Web-based teleconsultation has been positive. Users’ behaviors and perceptions toward the application are encouraging. They actively use the service and perceive it as helpful, and specialists are pleased to share their knowledge. We believe it would be worth the effort to expand and encourage the use of Doctor Chat in distant areas of Colombia and Latin America, as well as homologous applications in other developing countries.

**Figure 4:**
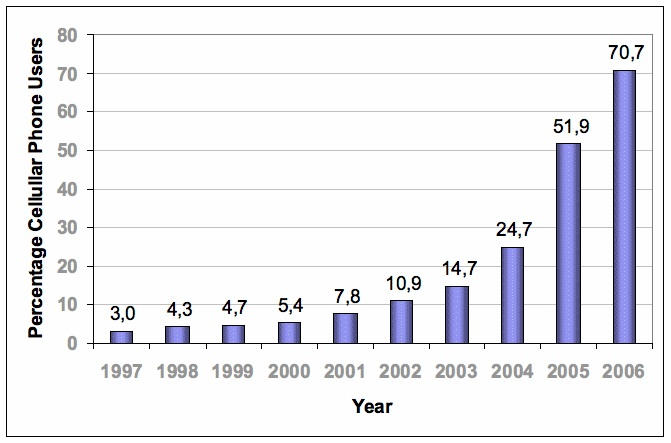
Penetration of mobile phones in Colombia [33,34]

Because “areas...most likely to benefit from telemedicine are those least likely to afford it or to have the requisite communications infrastructure” [32], methods to ensure equitable access to health care for sections of the population without connectivity are pivotal; however, ongoing SGSSS mechanisms to improve such access are slow in Colombia, and it is unlikely that short-term results are to be seen. As connectivity expands, teleconsulting could derive important and rapid impacts, especially for remote populations. In the meantime, simple interventions using cellular phones need to be considered, such as short messaging services (SMS). By observing the exponential increase in the penetration of mobile phones in the country over the past years (Figure 4), it could be concluded that these types of approaches might be where the highest potential for health care information delivery resides in the short term; however, mobile phone market oligopoly-related constraints (only three enterprises control the mobile telephony market of Colombia) make this difficult, and control over prices and access to infrastructure need to be resolved before health-related mobile phone consultations can be feasible.

Lastly, we envision Doctor Chat’s future development heading toward supporting isolated health care professionals in remote areas of Colombia. Efforts made by Swinfen et al [35,36] and Wootton [37] have shown that a low-cost approach to asynchronous teleconsultation for health care is helpful to referring doctors in rural areas and of benefit to their patients (see also the report of this group in this Theme Issue [38]). We are cognisant, however, that before implementing these developments, issues such as the Internet’s penetration and reliability, certain medico-legal issues, and assessment of the quality of medical consultants need to be addressed.

### Conclusion

The implementation of a Web-based teleconsulting service in Colombia constituted an innovative way to improve community access to health care and information and encouraged open and explicit discussion. Extending the service to underserved areas could improve access to health services and health information and could potentially improve economic indicators such as waiting times for consultations and the rate of pregnancy among teenagers; however, cultural, infrastructural, and connectivity barriers must be resolved before successful implementation can derive population-wide positive impacts. Taking into account the rapid growth and the high penetration of cellular phones in Colombia, making use of this resource could positively impact health care information delivery in the short term.

### Limitations

This research presents many limitations. First, the data analysis required all queries to be subjectively classified into only one of the three schemes described. The classification imposes a categorization bias as there were some multidisciplinary requests. Forthcoming evaluations of Doctor Chat will consider alternative schemes of classification to alleviate this problem. Second, the rural and remote populations for which Doctor Chat was created could not be evaluated. Aside from the lack of marketing and publicity of the site, factors such the low levels of Internet connectivity or the lack of access to computers may have played a major role. Further analysis will aim to address the service’s impact in remote areas. Third, Doctor Chat was not a secure application, and although major efforts have been put in place to provide a high-quality service, issues regarding confidentiality and safety of the users’ information need to be resolved. Last, in spite of the potential of the teleconsulting service to improve access to underserved populations, national and institutional infrastructure need to be extended before its diffusion and implementation on a national or regional scale will be feasible.

## Abbreviations

SGSSS: General Social Security System for Health (Sistema General de Seguridad Social en Salud
WHO: World Health Organization
